# Antioxidant properties of bioactive metabolites produced by lactic acid bacteria isolated from tropical fruits

**DOI:** 10.1007/s42770-026-02063-y

**Published:** 2026-08-25

**Authors:** Giselle Santos Silva, Kayque Ordonho Carneiro, Emília Maria França Lima, Svetoslav Dimitrov Todorov

**Affiliations:** 1 ProBacLab, Laboratório de Microbiologia de Alimentos, Departamento de Alimentos E Nutrição Experimental, Food Research Center, Faculdade de Ciências Farmacêuticas, Universidade de São Paulo, São Paulo, Brasil; 2 CISAS- Center for Research and Development in Agrifood Systems and Sustainability, Instituto Politécnico de Viana Do Castelo, Viana Do Castelo, Portugal

**Keywords:** Lactic acid bacteria, Probiotics, Tropical fruits, Natural antioxidants, Food safety

## Abstract

Lactic acid bacteria (LAB) have been widely used in the production of fermented foods since the early stages of civilization, playing a key role in the development of sensory characteristics and in food preservation. In recent years, scientific interest in these microorganisms has increased due to their potential health benefits, including their contribution to the balance of intestinal microbiota and the strengthening of the immune system. Moreover, the exploration of the potential of these microorganisms and their metabolites as antioxidant and antimicrobial agents in the food industry remains limited, particularly when they are isolated from tropical fruits. The present study aimed to identify and characterize LAB isolated from Brazilian fruits. The isolated strains were evaluated for safety through analyses of hemolytic activity, production of biogenic amines, mucin degradation, antibiotic susceptibility or resistance, and enzyme production. Genetic analyses were also performed to investigate the presence of virulence genes. Strains considered safe were further subjected to stability and survival assays under simulated conditions of the gastrointestinal tract and oral cavity, as well as to evaluations of cell surface hydrophobicity and extraction of exopolysaccharides (EPS), in order to identify compounds associated with antioxidant activity. A total of thirteen LAB isolates were obtained from tropical fruits, all of which demonstrated potential for application as natural antioxidants. These findings contribute to the selection of putative probiotic strains isolated from fruits of the Brazilian biome, such as blackberry, cashew, cherry of Rio Grande (*cereja-do-Rio-Grande*), guava, red lemon, and pitanga, with promising prospects for safe use related to their antioxidant properties.

**Supplementary Information:**

The online version contains supplementary material available at 10.1007/s42770-026-02063-y.

## Introduction

Microbiology establishes a critical interface between sustainability, health, and food safety. Its applications highlight that the use of microbial diversity represents an important strategy for improving quality of life, preserving ecosystems, and developing more resilient systems to address contemporary challenges. Lactic acid bacteria (LAB) are Gram-positive microorganisms, with rod- or cocci-shaped morphology, facultative anaerobic metabolism, and high tolerance to acidic environments. They belong to the phylum *Firmicutes* and the order *Lactobacillales* and are characterized by the absence of motility and spore formation. Their main defining feature is their metabolic ability to convert carbohydrates into lactic acid as the primary product of carbohydrates metabolisms, which has supported their use for millennia as starter cultures in food fermentation processes, contributing to preservation and sensory properties [1].

LAB are also capable of producing a wide range of bioactive metabolites, including organic acids, hydrogen peroxide, carbon dioxide, diacetyl, bacteriocins, exopolysaccharides (EPS), enzymes, and peptides. These compounds play a significant role in food biopreservation and represent promising alternatives to conventional antibiotics, especially in the context of increasing antimicrobial resistance [2, 3]. In addition, many of these metabolites exhibit antioxidant activity, which is essential for neutralizing reactive species such as reactive oxygen species (ROS), whose accumulation is associated with oxidative stress and the development of chronic diseases, including cancer, cardiovascular disorders, and neurodegenerative conditions [3, 4]. The antioxidant capacity of LAB is fundamental both for their own survival and for their ecological and functional roles. Oxidative stress, resulting from the accumulation of ROS, can damage essential cellular components such as DNA, proteins, and lipids. To counteract these effects, LAB synthesize antioxidant compounds such as EPS, bioactive peptides, and organic acids, which protect cellular structures and support their adaptation to diverse environments, including fermented foods, plant surfaces, and the gastrointestinal tract [5].

Despite advances in this field, studies focusing on LAB isolated from Brazilian tropical fruits remain limited. Given the high biodiversity of Brazilian biomes, tropical fruits represent promising ecological niches for the discovery of novel microorganisms with biotechnological potential. Many of these fruits are traditionally used by local populations for therapeutic purposes, suggesting the presence of functional compounds and microbial communities that remain underexplored [6]. Beyond their role in microbial survival, LAB antioxidant activity also influences the biochemical stability of fruits and derived products. By neutralizing ROS and reducing oxidative reactions, LAB can delay lipid oxidation, pigment degradation, and enzymatic browning, processes commonly observed in fruits rich in moisture and phenolic compounds. This effect is particularly relevant in fruits such as açaí and jabuticaba, which contain oxidation-sensitive compounds like anthocyanins and unsaturated lipids. Consequently, LAB contributes to color retention, sensory stability, and extended shelf life in fermented or minimally processed fruit products, while also enabling the biotransformation of polyphenols into more stable or bioactive forms [7].

From an ecological perspective, LAB antioxidant capacity can influence microbial dynamics on fruit surfaces by reducing oxidative stress in the microenvironment, thereby favoring their own growth while limiting the proliferation of oxidation-sensitive microorganisms. Although they do not directly participate in fruit maturation or plant defense, LAB contributes to postharvest stability by restricting the growth of spoilage microorganisms. This highlights their potential as natural stabilizing agents in fruit ecosystems and as strategic components in the development of functional foods derived from Brazilian biodiversity [8].

Additionally, LAB plays an important role in protecting the host organism against oxidative stress. Various strains possess enzymatic and non-enzymatic antioxidant systems, including superoxide dismutase, glutathione, and NADH oxidase, as well as the ability to metabolize phenolic compounds and produce EPS. When present in the gastrointestinal tract or consumed through fermented foods, LAB contribute to redox balance by neutralizing ROS, modulating antioxidant signaling pathways such as Nrf2–Keap1, and increasing the bioavailability of dietary polyphenols. Experimental evidence indicates that these mechanisms are associated with reduced oxidative stress markers, improved immune function, and enhanced resistance to inflammatory conditions, reinforcing the relevance of LAB in human and animal health [9, 10].

EPS production is closely associated with LAB antioxidant mechanisms and plays a key role in both microbial protection and functional performance. These biopolymers form a protective barrier around bacterial cells, reducing exposure to ROS and other oxidative agents. In addition, EPS can directly scavenge free radicals and interact with phenolic compounds, stabilizing them and modulating their bioavailability. This interaction enhances antioxidant activity in the gastrointestinal tract, contributing to mucosal protection and redox balance. Therefore, EPS not only strengthen microbial defense systems but also expand the functional potential of fermented foods, particularly those derived from plant sources rich in phenolic compounds [11].

In this context, investigating the microbiota associated with Brazilian tropical fruits may significantly contribute to the identification of novel probiotic strains and bioactive metabolites with applications in food safety, nutrition, and human health. Thus, the aim of this study was to isolate and identify LAB from Brazilian tropical fruits and to evaluate their microbiological safety, beneficial properties, antioxidant potential, and antimicrobial activity against foodborne pathogens.

## Materials and methods

### Fruits sampling

Cashew (*Anacardium occidentale*), guava (*Psidium guajava*) and pineapple (*Ananas comosus*) fruits were purchased from commercial establishments in the city of São Paulo, SP, Brazil. The remaining fruits, blackberry (*Morus* spp.), cherry of the Rio Grande (*Eugenia involucrata*), lemon (*Citrus* spp.), pitanga (*Eugenia uniflora*), and mango (*Mangifera indica*), were manually collected from areas located in the southern and western zones of São Paulo, SP, Brazil. Sampling was carried out in urban environments, and the samples were transported to the laboratory under aseptic conditions for subsequent processing.

### Screening, isolation, and identification of LAB

For microbial isolation, the collected fruits were initially weighed and homogenized in sterile saline solution (0.85% w/v) using a *Stomacher* device (Smasher, AES Laboratories, Ames, IA, USA), operating at 200 rpm for 2 min. Subsequently, serial dilutions were prepared at a 1:10 ratio. Aliquots of the dilutions were spread onto MRS (BD Difco, Detroit, MI, USA) supplemented with at 2% agar (w/v). The plates were incubated in an inverted position at 37 °C for 24–48 h. After incubation, isolated colonies were selected and inoculated into MRS broth, followed by incubation under conditions, as previously described.

The resulting cultures were stored at - 20 °C in MRS medium supplemented with 20% (w/v) glycerol until further analyses. Preliminary characterization of the isolates included Gram staining, microscopic examination, colony morphology observation, and catalase testing, as described by de Vos et al. [12].

### Safety assessment of strains

#### Hemolytic activity

The ability of the strains to induce erythrocyte lysis was evaluated according to the recommendations of Arellano-Ayala et al. [13]. Cultures of the strains of interest, as well as positive controls (*Listeria monocytogenes* ATCC 7644 and *Bacillus cereus* ATCC 11778) and a negative control (*Lactiplantibacillus plantarum* ST8SH), were streaked onto blood agar plates (Laborclin, SP, Brazil) and incubated at 37 °C for 16–24 h. After incubation, plates were observed and hemolytic activity was classified as follows: β-hemolysis (complete hemolysis), characterized by clear halos surrounding the colonies,α-hemolysis (partial hemolysis), indicated by greenish halos around the colonies; and γ-hemolysis, characterized by the absence of hemolysis. Experiments were performed in triplicates on two independent occasions.

#### Gelatinase production

The ability of the strains to hydrolyze gelatin was evaluated as described by Valledor et al. [14]. The strains were inoculated into MRS medium supplemented with 3% gelatin and incubated at 37 °C for 18 h. Subsequently, the tubes were refrigerated at 4 °C for 5 h. *B. cereus* ATCC 11778 was used as a positive control, and *Lpb. plantarum* ST8SH as negative control. Gelatin hydrolysis was determined by the liquefaction of the medium after refrigeration. Experiments were performed in duplicates on two independent occasions.

#### Proteolytic activity

The proteolytic activity of the isolates was determined according to Fugaban et al. [15]. Cultures grown for 18 h were transferred to MRS agar plates supplemented with 20% skim milk (Molico, Brazil). The plates were incubated at 37 °C for 24 h. Proteolytic activity was evaluated by the presence of clear zones surrounding the growing colonies, indicating protein degradation in the medium. Strains from the ProBacLab laboratory culture collection were used as controls. Experiments were performed in duplicates on two independent occasions.

#### Antibiotic susceptibility

Antimicrobial susceptibility was determined using the agar diffusion method, in accordance with the guidelines established by the Clinical and Laboratory Standards Institute (CLSI). The selection of antibiotics was based on the recommendations of the European Food Safety Authority (EFSA) [16], including: streptomycin (10 µg/disc), gentamicin (10 µg/disc), tetracycline (30 µg/disc), clindamycin (2 µg/disc), erythromycin (10 µg/disc), kanamycin (30 µg/disc), tylosin (60 µg/disc), nalidixic acid (30 µg/disc), vancomycin (30 µg/disc), and ampicillin (10 µg/disc). Studied strains were incorporated into the MRS supplemented with 2% agar and after solidification, antibiotics disks were placed on the surface and plates incubated for 24 h at 37 ºC. Pleats were observed for formation of inhibitory zones, expressed in millimeters inhibitory zones around placed discs. Results were interpreted according to the recommendations form Charteris et al. [17]. Experiments were performed in triplicates on two independent occasions.

#### Mucin degradation

The mucin degradation assay was performed as proposed cording to Abe et al. [18]. MRS medium supplemented with 1.5% agar (w/v), 0.3% HGM (w/v) (commercially obtained porcine gastric mucin, Sigma Aldrich) and with or without the addition of 1% glucose (w/v) was applied. Ten microliters of each bacterial culture (previously activated in MRS broth at 37 °C for 24 h) were placed on the surface of the plates and incubated in duplicate under anaerobic and aerobic conditions for 72 h at 37 °C. The colonies formed were stained with 0.1% black starch (w/v) in 3.5 M acetic acid for 30 min and then washed with 1.2 M acetic acid. The mucin lysis zone (discolored halos) around the colonies was served as evidence for mucin degradation.

### Differentiation and partial sequencing of the 16S rRNA gene of selected strains

Selected isolates were cultivated in MRS medium at 37 °C for 24 h. Genomic DNA was extracted using the ZR Fungal/Bacterial DNA Kit (Zymo Research, Irvine, CA, USA), according to the manufacturer’s instructions. DNA concentration and purity were assessed using a NanoDrop spectrophotometer (SpectrostarNano, Ortenberg, Germany).

For the initial differentiation of isolates, the rep-PCR technique was employed using the (GTG)₅ primer, as described by Choi et al. [19]. PCR reactions were performed in a Veriti 96 thermal cycler (Applied Biosystems, Thermo Fisher Scientific, Waltham, MA, USA) under the following conditions: initial denaturation at 95 °C for 5 min, followed by 30 cycles of denaturation at 95 °C for 30 s, annealing at 40 °C for 30 s, and extension at 65 °C for 8 min, with a final extension step at 65 °C for 16 min. Amplified products were separated by electrophoresis on 1.5% (w/v) agarose gels, stained with SYBR^®^ Safe DNA Gel Stain (Thermo Fisher Scientific), and visualized using a Bio-Rad gel documentation system (Hercules, CA, USA).

For taxonomic identification of representative strains from each cluster, partial sequencing of the 16S rRNA gene was performed following amplification with universal primers 27 F (5′-AGA GTT TGA TCC TGG CTC AG-3′) and 1492R (5′-GGT TAC CTT GTT ACG ACT T-3′) [19]. PCR reactions were carried out in a Veriti 96 thermal cycler using the following program: initial denaturation at 95 °C for 7 min, followed by 35 cycles consisting of denaturation at 95 °C for 1 min, annealing at 58 °C for 1.5 min, and extension at 72 °C for 2.5 min, with a final extension step at 72 °C for 5 min. Amplicons were separated by electrophoresis on 1% (w/v) agarose gels, stained with SYBR^®^ Safe DNA Gel Stain and visualized using a Molecular Imager® Gel Doc™ XR system (Bio-Rad). Sequencing was performed using the Sanger method at the Center for the Study of the Human Genome, Institute of Biomedical Sciences, University of São Paulo (USP), employing the BigDye Terminator v3.1 Cycle Sequencing Kit. The obtained sequences were analyzed using Sequencing Analysis 7.0 software (Base Caller KB™) and subsequently compared with sequences deposited in the National Center for Biotechnology Information (NCBI) database using the BLAST tool for taxonomic identification.

### Presence of beneficial and virulence genes

The selected strains were evaluated for the presence of genes associated with beneficial functions and virulence factors. For the detection of genes encoding adhesion-related proteins (*mapA* and *mub*) and elongation factor (*EF-Tu*), specific primers were used in PCR assays, as described by Todorov and Dicks [20]. The *in vitro* screening of key genes involved in the folate biosynthesis pathway (*folP, folE, folK, folQ, pabB,* and *pabC*) was conducted following the methodology described by dos Santos et al. [21]. The presence of genes related to bacteriocin production, including nisin and pediocin PA-1, was investigated by PCR following previously described protocols [22–26].

In parallel, the strains were screened for the presence of virulence genes, including *gelE, hyl, asa1, esp, cylA, efaA,* and *ace*, as well as genes associated with vancomycin resistance (*vanA, vanB, vanC, vanD, vanE, vanG*) and the mobile genetic element *IS16*. Additionally, genes encoding amino acid decarboxylases (*hdc1, hdc2, tdc,* and *odc*), previously described in streptococci, were also investigated. PCR reactions were carried out according to protocols described by Martin-Platero et al. [27], de las Rivas et al. [28], Vankerckhoven et al. [29].

Amplified products were separated by agarose gel electrophoresis (1.5–2.0% w/v) in 0.5 × TAE buffer, stained with SYBR^®^ Safe DNA Gel Stain, and visualized using a gel documentation system. All primers used in this study are presented in Supplementary Table 2, along with the results obtained for each gene investigated.

### Hydrophobicity

The ability of the strains to adhere to hydrocarbons, as a measure of cell surface hydrophobicity, was determined according to the method of Vinderola and Reinheimer [30], with some modifications. Cells from each studied strain were harvested at the stationary phase (10,000 × g, 5 min, 4 °C), washed twice with 50 mM phosphate buffer (pH 6.5), and resuspended in the same buffer. Three milliliters of the cell suspension, adjusted to an OD₅₆₀_nm_ of 1.0 with buffer, were mixed with 0.6 mL of *n*-hexadecane and vortexed for 120 s. The two phases were allowed to separate for 1 h at 37 °C. The aqueous phase was carefully removed, and the optical density was measured at 560 nm.

The percentage of cell surface hydrophobicity was calculated as [(A₀ − A₁)/A₀] × 100, where A₀ and A₁ represent the OD values before and after extraction with *n*-hexadecane, respectively. All assays were performed in quadruplicate to ensure greater reliability and statistical robustness of the results.

### Evaluation of antioxidant production

For the following assays, bacterial cultures were grown in MRS broth at 37 °C for 24 h. Cells were collected by centrifugation (3,000 × g, 10 min, 20 °C), resuspended in PBS (Lonza, Walkersville, MD, USA), and adjusted to approximately 11 log CFU/mL based on comparison with McFarland standards. Subsequently, 0.5 mL of the suspension was centrifuged (10,000 × g, 15 min, 20 °C), and the pellet was resuspended in 1 mL of PBS.

#### Determination of DPPH radical scavenging activity (2,2-diphenyl-1-picrylhydrazyl)

For this evaluation, experimental procedures described by Li et al. [31] and Duz et al. [32] were followed. Fresh cells from each culture were prepared as previously described. Then, 1 mL of the cell suspension in PBS was mixed with 1 mL of freshly prepared DPPH solution (0.05 mM in ethanol), and the samples were incubated in the dark for 1 h.

For the blank, the cell suspension was replaced with PBS. A solution of 1 mg/mL ascorbic acid (Sigma-Aldrich) in distilled water was used as a positive control. Supernatants from all samples were obtained by centrifugation (10,000 × g, 10 min, 20 °C) and analyzed spectrophotometrically at 517 nm.

The DPPH radical scavenging activity (%) of the tested strains was calculated according to Li et al. [31] and Duz et al. [32], as follows:

$$\mathrm{R}\mathrm{a}\mathrm{d}\mathrm{i}\mathrm{c}\mathrm{a}\mathrm{l}\;\mathrm{s}\mathrm{c}\mathrm{a}\mathrm{v}\mathrm{e}\mathrm{n}\mathrm{g}\mathrm{i}\mathrm{n}\mathrm{g}\;\mathrm{a}\mathrm{c}\mathrm{t}\mathrm{i}\mathrm{v}\mathrm{i}\mathrm{t}\mathrm{y}\;({\%}) = \{1-[({\mathrm{A}}_{\mathrm{s}\mathrm{a}\mathrm{m}\mathrm{p}\mathrm{l}\mathrm{e}}-\mathrm{A}\_\mathrm{b}\mathrm{l}\mathrm{a}\mathrm{n}\mathrm{k}\;\mathrm{c}\mathrm{o}\mathrm{n}\mathrm{t}\mathrm{r}\mathrm{o}\mathrm{l}) / \mathrm{A}\_\mathrm{b}\mathrm{l}\mathrm{a}\mathrm{n}\mathrm{k}]\} \times 100$$

where: *A_sample* = sample; *A_blank control* = PBS solution; *A_blank* = PBS solution with DPPH.

#### Determination of Fe^2^⁺ chelating activity

For this evaluation, the method described by Decker and Welch [33] was followed. Briefly, 1 mL of previously prepared intact cells, resuspended in PBS, was mixed with 0.05 mL of a 2 mM FeCl₂ solution. To initiate the reaction, 0.2 mL of 5 mM ferrozine was added, and the samples were incubated in the dark for 10 min. A 1 mg/mL ascorbic acid solution was used as a positive control. After incubation, the supernatants of the samples were obtained by centrifugation (10.000 × g, 10 min, 20 °C) and analyzed spectrophotometrically at 562 nm to determine the reduction in optical density.

The chelating activity was calculated as follows:

$$\mathrm{F}\mathrm{e}\mathrm{r}\mathrm{r}\mathrm{o}\mathrm{u}\mathrm{s} \mathrm{i}\mathrm{o}\mathrm{n}\;\mathrm{c}\mathrm{h}\mathrm{e}\mathrm{l}\mathrm{a}\mathrm{t}\mathrm{i}\mathrm{n}\mathrm{g}\;\mathrm{a}\mathrm{c}\mathrm{t}\mathrm{i}\mathrm{v}\mathrm{i}\mathrm{t}\mathrm{y}\;({\%}) = [1-(\mathrm{O}\mathrm{D}\_\mathrm{s}\mathrm{a}\mathrm{m}\mathrm{p}\mathrm{l}\mathrm{e} / \mathrm{O}\mathrm{D}\_\mathrm{c}\mathrm{o}\mathrm{n}\mathrm{t}\mathrm{r}\mathrm{o}\mathrm{l})] \times 100$$

where: *OD_sample* = optical density of the sample; *OD_control* = optical density of the control solution.

#### Determination of hydroxyl radical scavenging activity

For this assay, the method described by Wang et al. [34] was followed. Intact cells, prepared as previously described, were used. Then, 1 mL of the cell suspension in PBS was transferred to glass test tubes containing 1 mL of brilliant blue solution (0.435 mM), 2 mL of FeSO₄ (0.5 mM), and 1.5 mL of H₂O₂ (3%, w/v). The mixtures were incubated at 37 °C for 1 h. A 1 mg/mL ascorbic acid solution (Sigma-Aldrich) was used as a positive control. After incubation, the samples were centrifuged (3,000 × g, 5 min, 20 °C), and the absorbance of the resulting supernatants was measured at 624 nm.

The hydroxyl radical scavenging activity (%) was calculated according to Wang et al. [34] as follows:

$$\mathrm{H}\mathrm{y}\mathrm{d}\mathrm{r}\mathrm{o}\mathrm{x}\mathrm{y}\mathrm{l}\;\mathrm{r}\mathrm{a}\mathrm{d}\mathrm{i}\mathrm{c}\mathrm{a}\mathrm{l}\;\mathrm{s}\mathrm{c}\mathrm{a}\mathrm{v}\mathrm{e}\mathrm{n}\mathrm{g}\mathrm{i}\mathrm{n}\mathrm{g}\;\mathrm{a}\mathrm{c}\mathrm{t}\mathrm{i}\mathrm{v}\mathrm{i}\mathrm{t}\mathrm{y}\;({\%}) = [({\mathrm{A}}_{1}-{\mathrm{A}}_{0}) / (\mathrm{A}-{\mathrm{A}}_{1})] \times 100$$

where: *A₀* = absorbance of the solution containing the sample; *A₁* = absorbance of the solution without the test sample; *A* = absorbance of the solution without the test sample and without the Fenton reaction system.

#### Determination of peroxide (Superoxide) radical scavenging activity

For the evaluation of peroxide radical (O₂⁻) scavenging activity, the method described by Wu et al. [35] was followed, using spectrophotometric analysis based on enhanced pyrogallol autoxidation. The superoxide anion radical was generated under alkaline conditions using a pyrogallol (1,2,3-benzenetriol) autoxidation system. Previously prepared cell suspensions (0.1 mL of microbial sample), adjusted to an optical density of 0.8, were mixed with 4.5 mL of Tris–HCl buffer (0.05 M, pH 8.2), and the suspension was incubated for 20 min at 25 °C in a water bath. Subsequently, 0.4 mL of pyrogallol solution (0.25 M, pre-warmed to 25 °C) was added, and the mixture was incubated for 4 min at 25 °C. The reaction was terminated by adding 0.1 mL of 1 M HCl. Absorbance was measured at 320 nm for calculation purposes. As a control, an equal volume of 50 mmol/L Tris–HCl buffer (pH 8.2) was used instead of the sample, while 1 mg/mL ascorbic acid and BHA were used as positive controls.

The peroxide radical scavenging activity (%) was calculated as follows:

$$\mathrm{P}\mathrm{e}\mathrm{r}\mathrm{o}\mathrm{x}\mathrm{i}\mathrm{d}\mathrm{e}\;\mathrm{r}\mathrm{a}\mathrm{d}\mathrm{i}\mathrm{c}\mathrm{a}\mathrm{l}\;\mathrm{s}\mathrm{c}\mathrm{a}\mathrm{v}\mathrm{e}\mathrm{n}\mathrm{g}\mathrm{i}\mathrm{n}\mathrm{g}\;\mathrm{a}\mathrm{c}\mathrm{t}\mathrm{i}\mathrm{v}\mathrm{i}\mathrm{t}\mathrm{y}\;\left({\%}\right)=[({\mathrm{A}}_{0}-{\mathrm{A}}_{1}) / {\mathrm{A}}_{0}] \times 100$$

where: *A₁* = absorbance of the samples; *A₀* = absorbance of the solution without the sample.

#### Determination of lipid peroxidation inhibition capacity

The lipid peroxidation inhibition capacity was determined using the method described by Hsu et al. [36] Fresh egg yolk (Difco) was supplemented with an equal volume of PBS (200 mM, pH 7.2), and the suspension was homogenized using a vortex. The resulting suspension was then diluted with PBS (egg yolk/PBS, 1:25, v/v). Subsequently, 1 mL of the egg yolk suspension was mixed with 0.5 mL of the microbial sample solution (adjusted to 11 log CFU/mL, as previously described), 1 mL of PBS, and 1 mL of iron sulfate (25 mM). The mixture was incubated at 37 °C for 15 min with agitation. After incubation, 1 mL of trichloroacetic acid (20%, w/v) was added, and the mixture was allowed to stand for 10 min, followed by centrifugation (3,000 × g, 10 min, 20 °C). Then, 2 mL of thiobarbituric acid (0.8%, w/v) was added to 3 mL of the supernatant, and the mixture was heated in a boiling water bath for 10 min and subsequently cooled to room temperature. The final supernatant was obtained by centrifugation (3.000 × g, 10 min, 20 °C), and the absorbance was measured at 532 nm (*A_sample*). The blank consisted of 0.5 mL of PBS replacing the sample (*A₀*).

The lipid peroxidation inhibition rate (%) was calculated as follows:

$$\mathrm{L}\mathrm{i}\mathrm{p}\mathrm{i}\mathrm{d}\;\mathrm{p}\mathrm{e}\mathrm{r}\mathrm{o}\mathrm{x}\mathrm{i}\mathrm{d}\mathrm{a}\mathrm{t}\mathrm{i}\mathrm{o}\mathrm{n}\;\mathrm{i}\mathrm{n}\mathrm{h}\mathrm{i}\mathrm{b}\mathrm{i}\mathrm{t}\mathrm{i}\mathrm{o}\mathrm{n} (\mathrm{\%}) = [({\mathrm{A}}_{0}-\mathrm{A}\_\mathrm{s}\mathrm{a}\mathrm{m}\mathrm{p}\mathrm{l}\mathrm{e}) / {\mathrm{A}}_{0}] \times 100$$

A 1 mg/mL ascorbic acid solution was used as a positive control.

### Evaluation of strain survival under gastrointestinal conditions

#### Evaluation of strain survival under simulated oral cavity conditions

To evaluate the effect of artificial saliva on the survival and viability of the studied LAB, the methodology described by Pytko-Polonczyk et al. [37] was followed. The strains were cultivated in 10 mL of MRS broth at 37 °C for 24 h. Cells were then harvested by centrifugation (4,000 × g, 5 min, 20 °C), washed twice with sterile saline solution (0.85% NaCl, w/v), and resuspended in artificial saliva solution containing: 25 mM KH₂PO₄, 24 mM Na₂HPO₄, 150 mM KHCO₃, 100 mM NaCl, 1.5 mM MgCl₂, 25 mM citric acid, and 15 mM CaCl₂.

The bacterial suspensions were incubated at 37 °C for 30 min. Representative samples were collected at the beginning of exposure (time zero) and at the end of the incubation period (30 min). Colony-forming units per milliliter (CFU/mL) were determined by serial dilution in sterile saline solution, followed by plating on MRS agar (2% w/v). Plates were incubated at 37 °C for 48 h for subsequent colony counting. Comparative analyses were then performed between CFU/mL counts before and after exposure to the artificial saliva model for all evaluated strains.

#### Evaluation of strain survival under stomach-duodenal passage simulation (SDPS) models

The survival of the strains under gastrointestinal conditions was evaluated using a simplified *in vitro* SDPS, adapted from Fugaban et al. [15], with modifications.

Bacterial cultures were initially grown in MRS broth (10 mL) for 24 h at 37 °C. Cells were then harvested by centrifugation (4,000 × g, 5 min, 20 °C), washed twice with sterile saline solution (pH 6.0; 0.85% NaCl, w/v), and resuspended in the original volume of saline adjusted to pH 2.5. The suspensions were homogenized and incubated for 60 min at 37 °C to simulate gastric conditions. Subsequently, 4 mL of sterile bile salt solution (10% bovine bile, w/v) and 17 mL of synthetic duodenal juice (pH 6.0; NaHCO₃ 6.4 g/L; KCl 0.239 g/L; NaCl 1.28 g/L) were added. The mixture was incubated for 2 h at 37 °C to simulate small intestinal conditions. For viability analysis, samples were collected at three stages: initial control (T_0_), post-gastric phase (T_1_), and post-intestinal phase (T_2_). Samples were subjected to serial dilutions in sterile saline solution and plated on supplemented MRS agar (2% w/v). After incubation at 37 °C for 48 h, colony-forming units (CFU/mL) were enumerated and compared to the initial population to determine survival rates.

### Evaluation of antioxidant activity before and after SDPS

The antioxidant activity of the studied strains was evaluated using the DPPH assay, comparing the results obtained before and after the SDPS models, in order to assess the influence of the simulated gastrointestinal environment on the antioxidant potential of the samples.

### Evaluation of the ability of strains to produce exopolysaccharides from different sugars

The ability of the strains to produce EPS was evaluated by inoculation in Petri dishes containing modified MRS medium, formulated with different carbon sources. Four types of sugars were used—glucose, sucrose, lactose, and fructose—resulting in four variations of the culture medium. Each strain was inoculated in triplicate plates containing MRS supplemented with each of the aforementioned sugars. The plates were incubated at 37 °C for 48 h. After the incubation period, EPS production was visually assessed based on the formation of mucous and/or viscous material on the surface of the medium, a characteristic indicative of the synthesis of these biopolymers [38].

EPS production was carried out according to the methodology described by Domingos-Lopes et al. [38] with modifications. The previously selected strains were previously cultured with a 1% inoculum in 50 mL of MRS broth and incubated at 37 °C for approximately 18–24 h. Subsequently, the culture was transferred to 1 L of modified MRS broth supplemented with 10% (w/v) of the tested sugars (glucose, fructose, sucrose, or lactose) and incubated for 48 h at 37 °C under fermentation conditions.

After the incubation period, the cells were removed by centrifugation (9,000 × g, 5 min, 4 °C). EPS was precipitated from the supernatant by the addition of two volumes of pre-chilled ethanol (4 °C), and the mixture was maintained under refrigeration for 48 h. Subsequently, centrifugation was performed to recover the precipitate (10,000 × g, 20 min, 4 °C). The obtained material was resuspended in Milli-Q water at room temperature. The precipitation step was repeated twice, and the EPS precipitates were dried under vacuum (Speedvac Concentrator, model SPD11V-115) to obtain purified EPS. The experimental procedure was conducted in quadruplicate to ensure an adequate amount of material for EPS yield determination.

### Evaluation of the antioxidant activity of EPS produced by selected strains

After drying the EPS produced by each strain, its antioxidant potential was evaluated using the DPPH radical scavenging assay, as described before. The EPS samples were compared with their respective producing strains in order to determine whether the antioxidant potential observed in the bacterial cells was associated with EPS production.

## Results

### Isolation, identification and safety of selected LAB

During the initial screening and isolation stage, more than 200 isolates were obtained from ten fruits: blackberry, cashew, cherry of Rio Grande, guava, red lemon, pitanga, banana, tangerine, mango, and pineapple. Among these, more than sixty isolates (Gram positive and catalase negative) were selected for subsequent studies. From this set, thirty isolates were excluded from further analysis based on the following phenotypic criteria: presence of α- or β-hemolysis. It is noteworthy that members of the genus *Bacillus* are catalase-positive, which explains why part of these isolates were excluded from subsequent analyses. LAB are generally characterized as Gram-positive, non-spore-forming, non-motile, and catalase-negative microorganisms, displaying either coccoid or rod-shaped morphology. In following evaluation, none of the evaluated isolates exhibited gelatinase or proteolytic activity. These preliminary assays were important for reducing the number of isolates that did not meet the objectives of the study, prioritizing microorganisms belonging to LAB with potential safe application.

It is important to emphasize that not all LAB can be considered safe or beneficial. Although some strains have Generally Recognized as Safe (GRAS) status, this classification is not universal and must be assigned individually to each strain following a thorough evaluation of safety-related properties, in accordance with the recommendations of the EFSA [16]. In this context, for applications such as probiotics or starter cultures, complete genome sequencing and detailed bioinformatic analysis are recommended, including structural characterization of bioactive compounds, to ensure that the use of such strains does not pose risks to human or animal health.

In the preliminary screening analysis, the antibiotic resistance and susceptibility profiles were also evaluated. Isolates showing resistance to more than three antimicrobial agents from different groups were excluded from further investigations. The aforementioned authority has established specific guidelines regarding the antibiotics to be tested for different groups of LAB. Additionally, vancomycin resistance was assessed in isolates with coccoid morphology, considering the possibility of later identification as *Enterococcus* spp., whose resistance to this antimicrobial represents a significant public health concern, particularly in nosocomial infections [16].

**Fig. 1:**
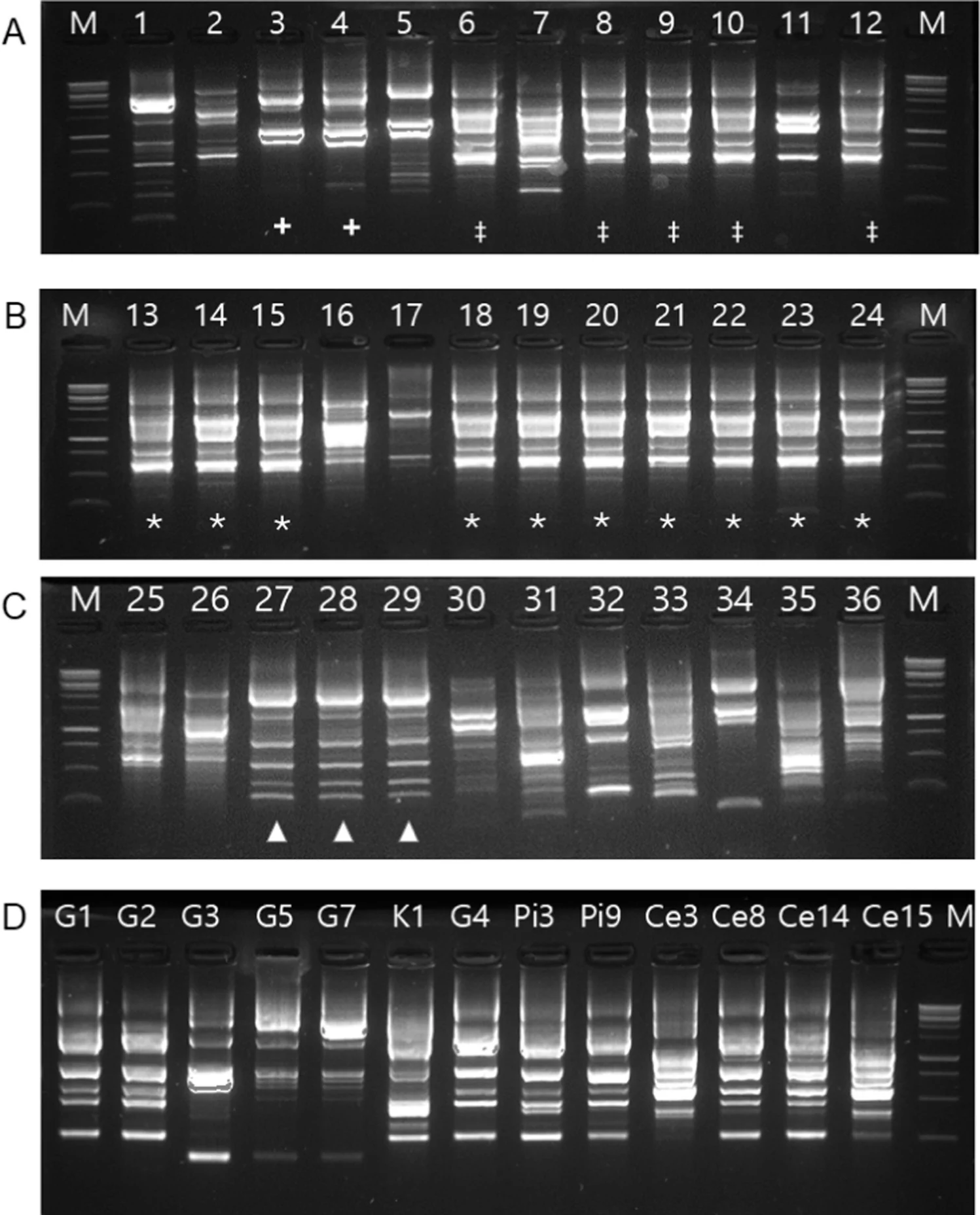
Differentiation of the isolates by rep-PCR based on the similarity of their banding patterns. Dendrograms showing the clustering of isolates are presented in panels (**A**–**C**), and a representative rep-PCR gel of the selected strains is shown in panel (**D**). **M**, 1 kb DNA ladder (Promega). Equal profiles were indicated by same symbols (+, ‡, *, ▲)

In the follow-up phase, 60 isolates with rod-shaped and coccoid morphology were selected for further investigation, all of which were preliminarily considered potentially safe based on the set of assays performed. In the final sequencing step, all submitted strains were identified as belonging to the LAB group. To differentiate the isolates of interest, rep-PCR profiling was performed. Isolates displaying identical banding patterns in agarose gel electrophoresis were considered genetically similar, and only one representative of each profile was retained for subsequent analyses (Fig. 1).

Subsequently, partial sequencing of the 16S rRNA gene was performed for taxonomic identification of representative strains from each cluster, following amplification with universal primers 27 F and 1492R, as described by [19].

The identification of the selected strains, their isolation fruits source, safety assessment and antibiotic susceptibility profiles are presented in Supplementary Table S1.

Additionally, the selected strains were evaluated for the presence of genes associated with beneficial functions and virulence factors. The primers used for PCR amplification and the detection of genes related to virulence, vancomycin resistance, adhesion, γ-aminobutyric acid (GABA) production, bile salt deconjugation, and biogenic amine production are presented in Supplementary Table 2.

### Functional characterization and antioxidant properties of the selected strains

**Fig. 2:**
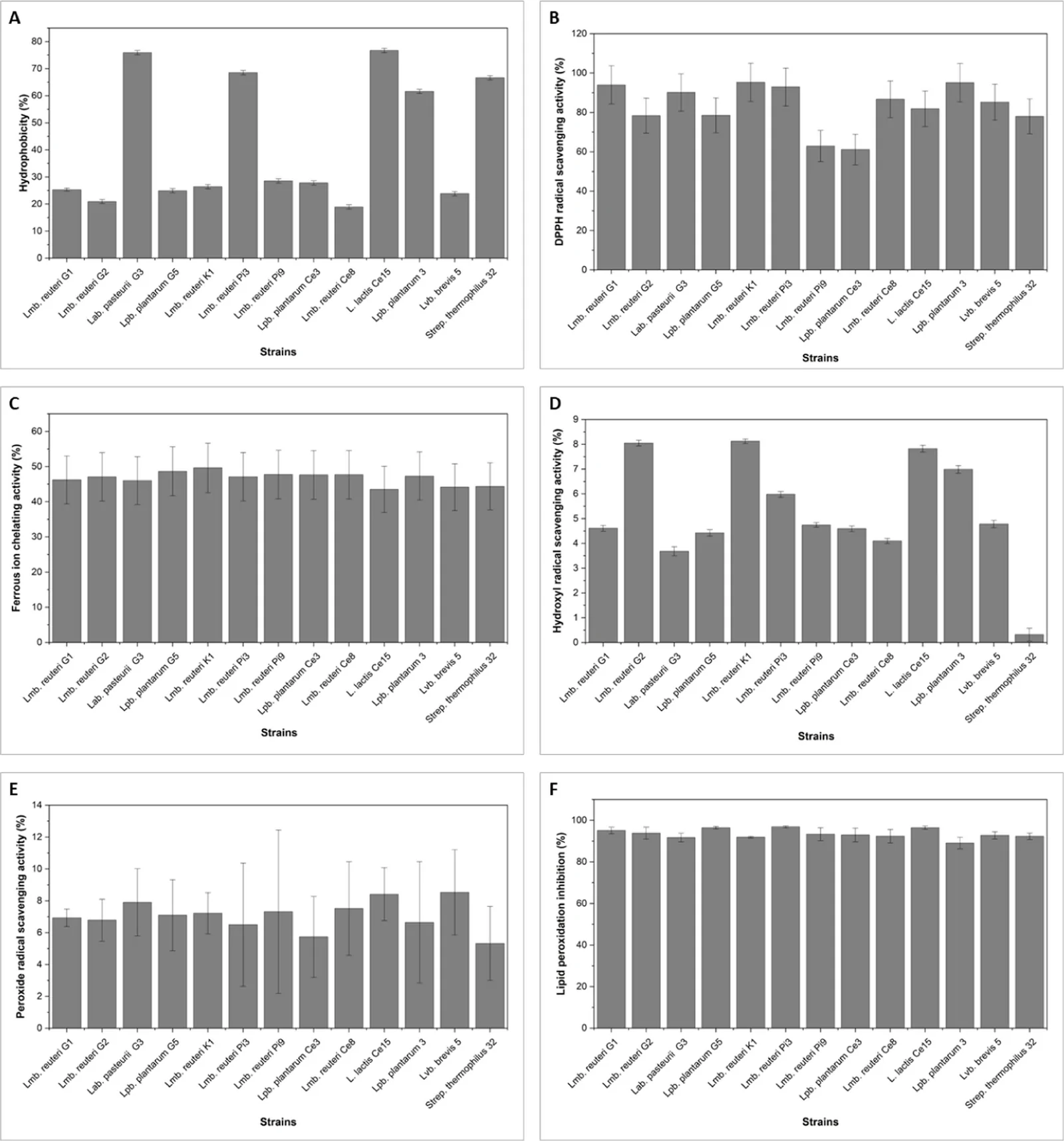
Functional characterization and antioxidant properties of the selected strains. (**A**) Cell surface hydrophobicity; (**B**) DPPH radical scavenging activity; (**C**) Ferrous ion (Fe^2^⁺) chelating activity; (**D**) Hydroxyl radical scavenging activity; (**E**) Peroxyl radical scavenging activity; and (**F**) Inhibition of lipid peroxidation. Results are expressed as the mean ± standard deviation

Regarding hydrophobicity, the strains *Lactococcus lactis* Ce15 and *Lactobacillus pasteurii* G3 exhibited higher hydrophobicity than the others, whereas the strain *Limosilactobacillus reuteri* Ce8 showed the lowest hydrophobicity (Fig. 2A).

The DPPH scavenging activity observed among the strains ranged from 95.29% for *Lmb. reuteri* K1 to 61.14% for *Lmb. reuteri* Ce3, indicating that antioxidant properties are strain-specific (Fig. 2B). Ferrous ion (Fe^2^⁺) chelating activity was similar among the selected strains, with values of approximately 45% for all isolates (Fig. 2C). Regarding hydroxyl radical scavenging activity, the strains exhibited variable but overall low levels, ranging from 0.32% for *Streptococcus thermophilus* 32 to 8.12% for *Lmb*. *reuteri* K1 (Fig. 2D). Peroxyl radical scavenging activity was also low among the selected strains, with the highest activity observed for *Levilactobacillus brevis* 5 (8.52%) (Fig. 2E). Lipid peroxidation inhibition activity was high for all selected strains, ranging from 89% for *Lpb. plantarum* 3 to 97% for *Lmb. reuteri* Pi3 (Fig. 2F).

The antioxidant properties observed exhibited a strain-specific character, varying among isolates even when belonging to the same microbial species. This variability highlights the need for individual evaluation of each strain regarding its functional potential.

The identified antioxidant attributes, combined with antimicrobial activity, represent technologically relevant characteristics for application in fermented food products. The simultaneous presence of these properties may contribute to reducing the use of chemical additives, meeting the growing consumer demand for more natural foods with lower levels of synthetic compounds.

The production of antimicrobial proteins, such as bacteriocins, together with antioxidant capacity, represents a promising scenario for the selection of new starter cultures with multiple beneficial properties. This approach expands the possibilities for biotechnological application of the studied strains, adding functional value and potentially enhancing the safety and stability of fermented foods.

### Evaluation of strain survival under simulated oral cavity conditions

**Fig. 3:**
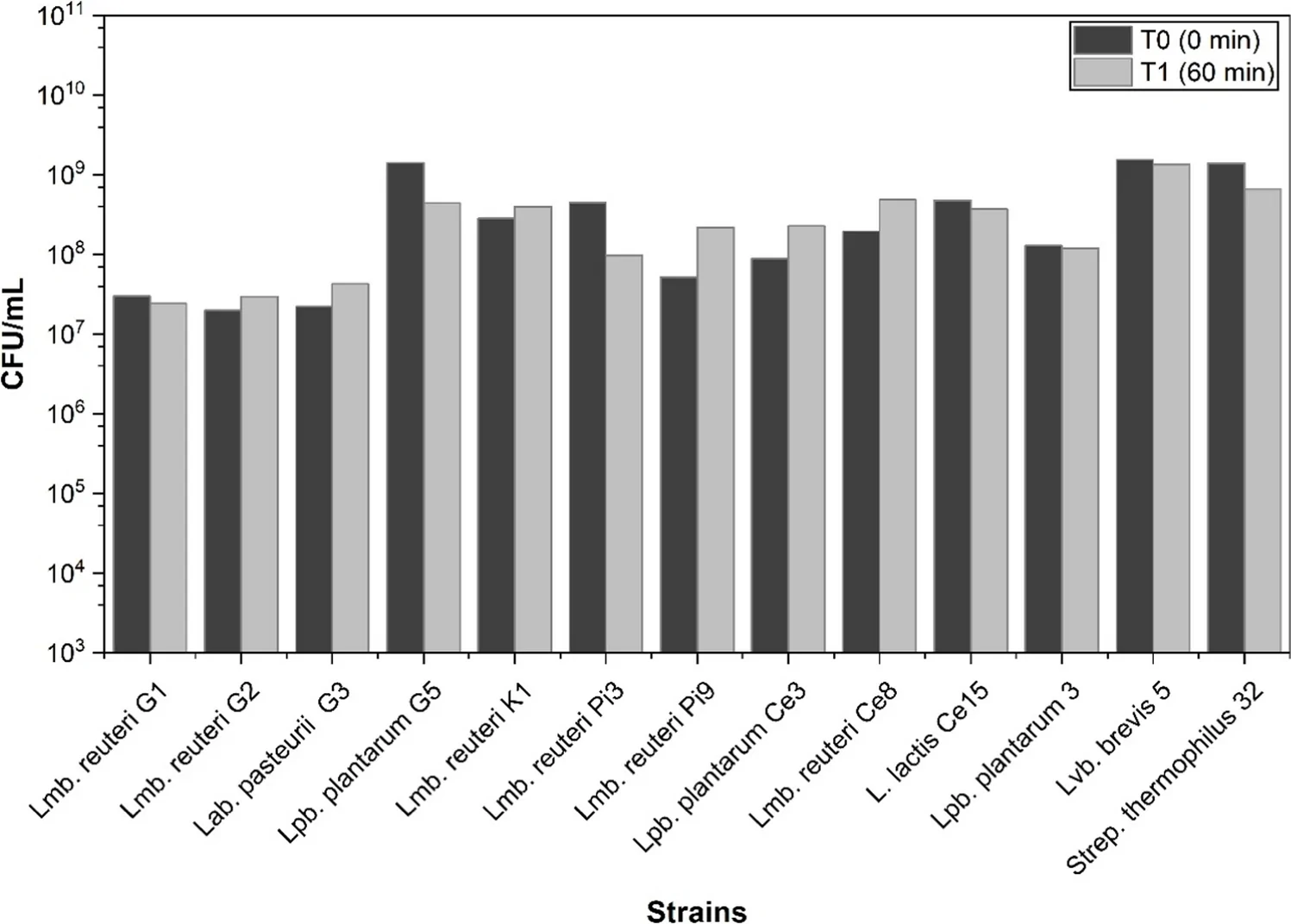
Analysis of the survival capacity of the strains under simulated oral cavity conditions. T₀ corresponds to the initial time point, at which the strain comes into contact with the simulated oral cavity environment. T₁ refers to the one‑hour exposure period of the strain to the environment that simulates oral conditions

The data obtained demonstrated that all evaluated strains exhibited a high survival capacity under simulated oral cavity conditions, with only slight variation between the initial cell counts and those observed at the end of the experiment. Notably, the strains *Lmb. reuteri* Pi9, *Lpb. plantarum* Ce3, and *Lmb. reuteri* Ce8 showed an increase in cell numbers after the experimental period, indicating that oral cavity conditions may represent a potentially favorable environment for the growth of LAB (Fig. 3).

### Evaluation of strain survival under SDPS

**Fig. 4:**
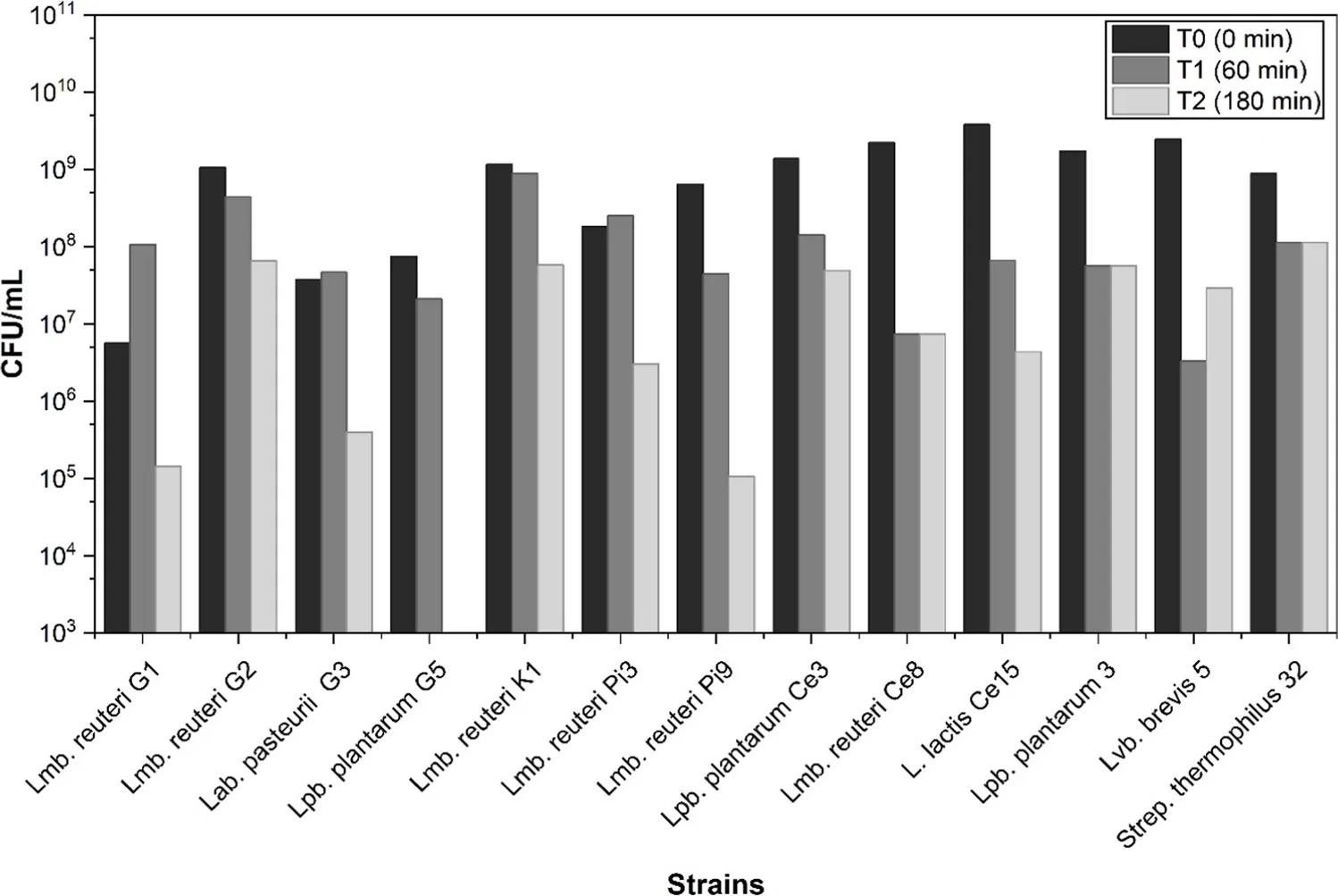
Analysis of the survival capacity of the strains under SDPS conditions. T₀ corresponds to the initial time point, at which the strain comes into contact with the simulated stomach environment. T₁ refers to the one‑hour exposure period of the strain to the environment that simulates gastric conditions, maintained at a constant temperature of 37 °C. T₂ corresponds to the period during which the strain remains for two hours in an environment that simulates duodenal conditions

As observed in Fig. 4, the strain *Lpb. plantarum* G5 did not exhibit survival under the simulated gastrointestinal passage conditions. In contrast, the strains *Lmb. reuteri* G2, *Lmb. reuteri* K1, *Lmb. reuteri* Pi3, *Lmb. reuteri* Pi9, *Lpb. plantarum* Ce3, *Lmb. reuteri* Ce8, *L. lactis* Ce15, *Lpb. plantarum* 3, *Lv. brevis* 5, and *Strep. thermophilus* 32 showed higher survival capacity under the simulated conditions compared to the other strains evaluated in this study.

Additionally, it was observed that all analyzed strains exhibited a reduction in antioxidant activity after simulation of gastrointestinal conditions. This result suggests that the gastrointestinal environment exerts a significant influence on the stability and/or functionality of bioactive compounds produced by the strains, potentially compromising their antioxidant activity after exposure to simulated digestive conditions.

### Evaluation of antioxidant activity of the studied strains before and after SDPS models

**Fig. 5:**
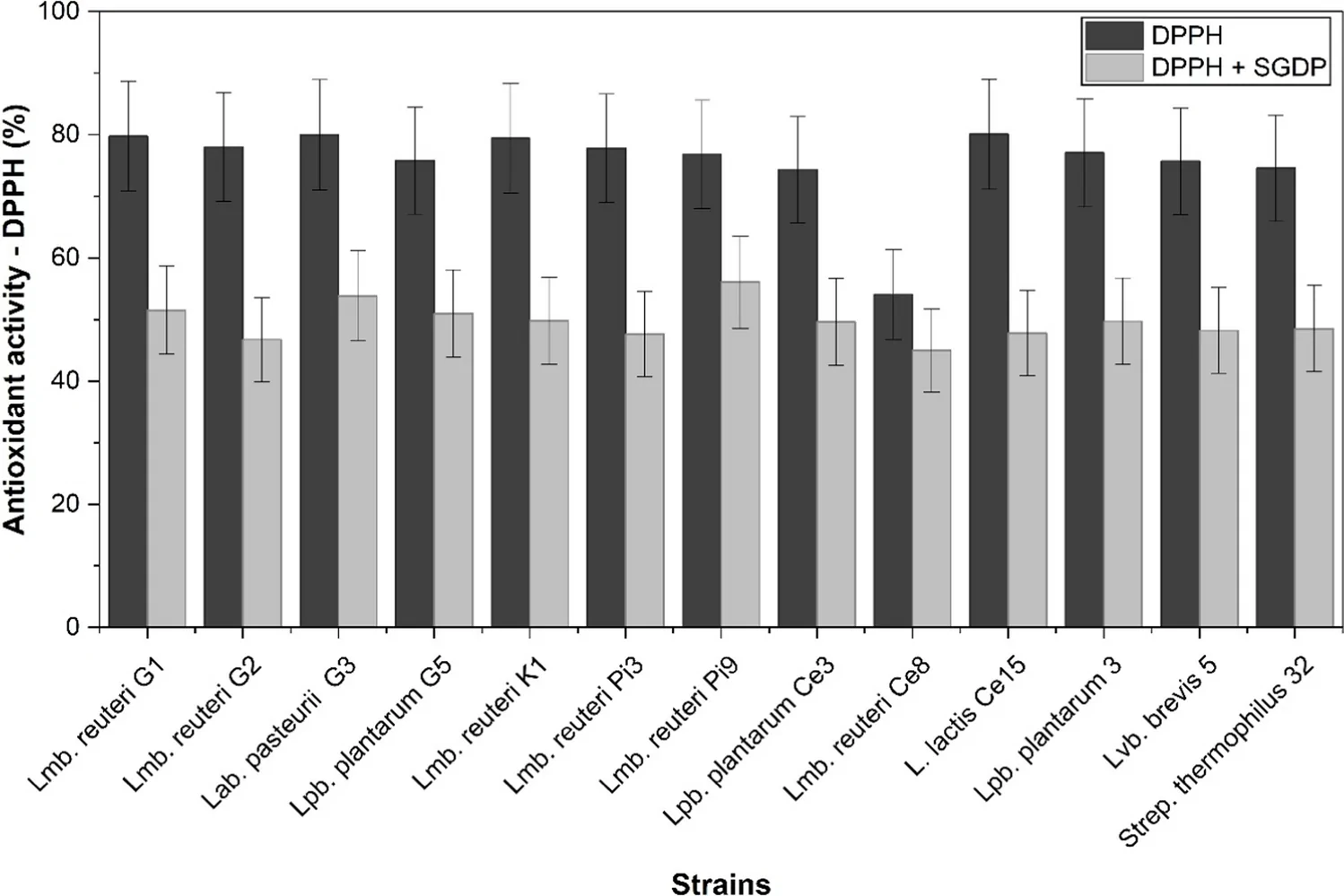
DPPH Oxidation Activity under SDPS conditions: with and without SDPS models. The strains were evaluated for antioxidant activity using the DPPH method, before and after the SDPS, with the aim of comparing antioxidant activity and assessing the influence of gastrointestinal tract conditions on this activity

The obtained data demonstrated that all evaluated strains exhibited a reduction in antioxidant activity after simulation of gastrointestinal conditions. This result indicates that the gastrointestinal environment exerts a direct influence on the stability and/or functionality of the bioactive compounds produced by the strains. However, the *Lmb. reuteri* Ce8 stood out by showing the lowest percentage reduction in antioxidant activity, indicating greater resistance to the simulated gastrointestinal conditions. This behavior suggests a higher potential for survival and maintenance of functional activity after passage through the digestive system, a characteristic relevant for probiotic applications (Fig. 5).

### Evaluation of the capacity of strains to produce EPS from different sugars

For the following assays, one representative strain from each bacterial species under study was selected in order to evaluate differences in microbial growth in the presence of four types of sugars: sucrose, fructose, glucose, and lactose.

**Fig. 6:**
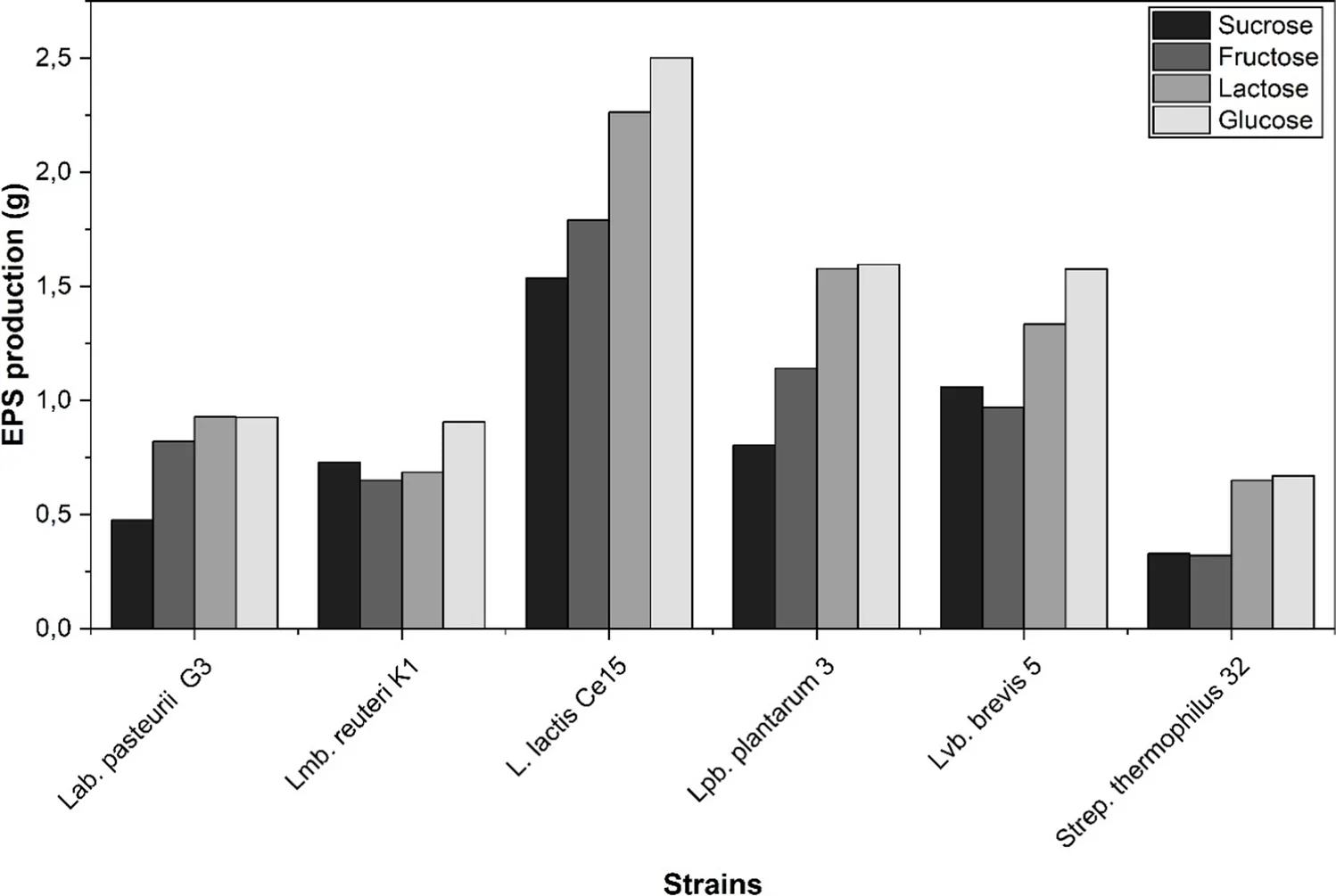
Analysis of the EPS‑producing capacity using four different types of sugars. T₀ corresponds to the initial time point, at which the strain comes into contact with the simulated oral cavity environment. T₁ refers to the one‑hour exposure period of the strain to the environment that simulates oral conditions

The evaluated strains showed higher utilization of glucose and lactose compared to sucrose and fructose. Furthermore, EPS production followed a similar trend, with enhanced performance observed when the microorganisms were cultivated in the presence of glucose and lactose (Fig. 6).

### Determination of antioxidant activity using the DPPH method: comparison between bacterial strains and EPS obtained from different sugars

**Fig. 7:**
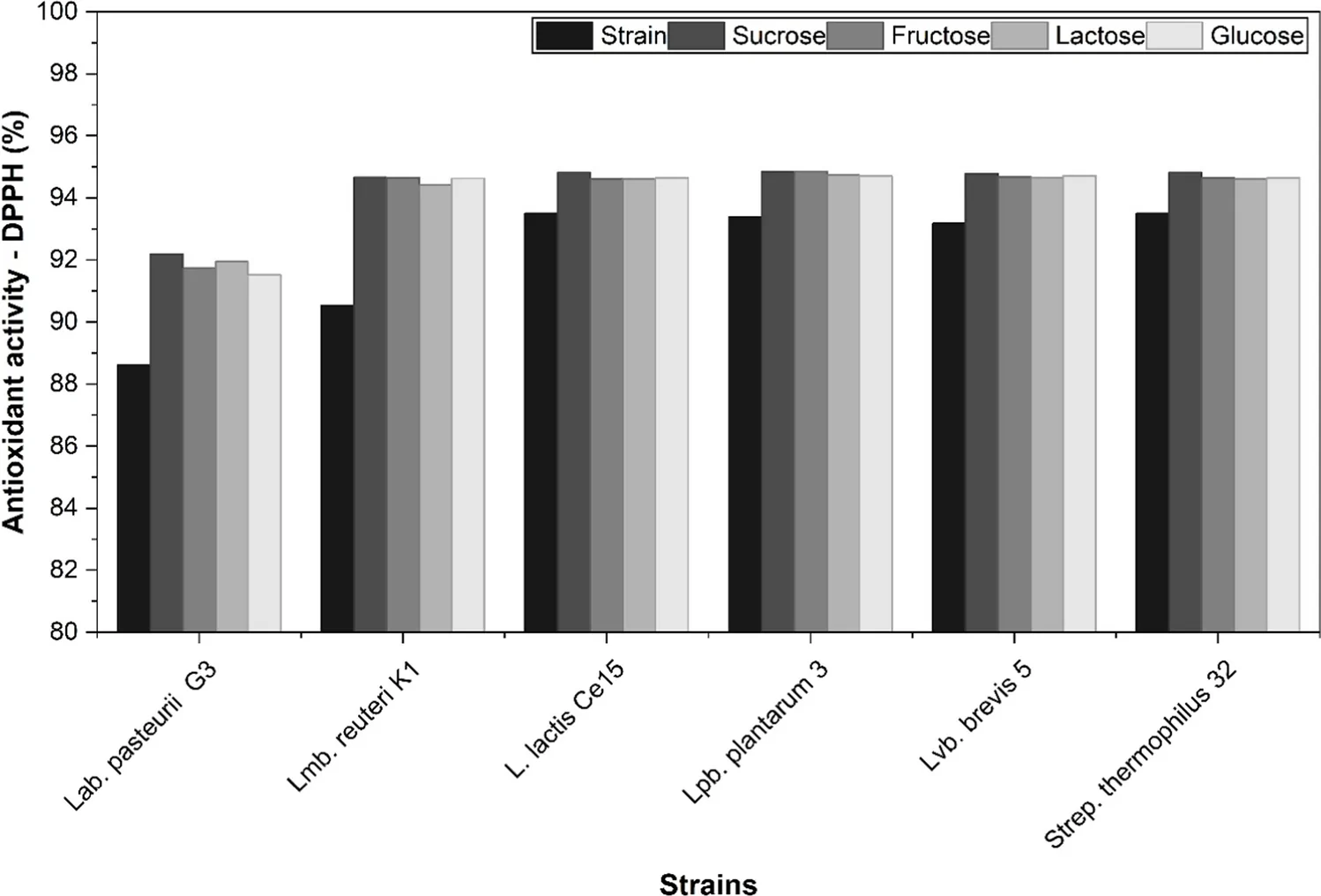
Determination of antioxidant activity using the DPPH method: comparison between the strain and the EPS obtained from different sugars*.* The first column for each strain corresponds to the oxidative activity of the strain itself (bacterial culture), while the remaining columns correspond to the EPS produced by the strain in the presence of each indicated sugar

In this assay, the antioxidant activity of each bacterial strain was evaluated and compared with the antioxidant activity of the EPS produced by the same strains, obtained through fermentation of different sugars (sucrose, fructose, glucose, and lactose). The results indicated that the EPS produced by each strain exhibited higher antioxidant activity compared to their respective bacterial cells, regardless of the type of sugar used in the fermentation process (Fig. 7).

## Discussion

The results obtained in this study demonstrate that tropical and subtropical fruits represent a relevant source of microorganisms with biotechnological potential, particularly LAB. The microbial diversity observed in these fruits can be explained by the chemical composition of these plant matrices, which generally exhibit high concentrations of fermentable sugars, organic acids, phenolic compounds, and various micronutrients capable of supporting the growth of fermentative microorganisms. Thus, natural environments such as fresh fruits constitute important ecological niches for the prospection of new microbial strains with potential applications in food, biotechnology, and health.

The initial screening of the isolated strains was based on classical microbiological characterization parameters, including Gram staining, catalase test, hemolytic activity, and gelatinolytic and proteolytic assays. The selected strains exhibited a phenotypic profile consistent with LAB, mainly characterized as Gram-positive, catalase-negative, and non-spore-forming. These characteristics are consistent with the traditional description of this microbial group, which includes widely used genera in food microbiology such as *Lactobacillus*, *Lactiplantibacillus*, *Limosilactobacillus*, and *Lactococcus*.

The absence of hemolytic activity in the selected strains represents an important preliminary indicator of microbiological safety. Hemolysis is frequently associated with virulence factors and is considered an essential criterion in the evaluation of microorganisms intended for food or probiotic applications [13]. Similarly, the absence of gelatinase and proteolytic activity also suggests that the analyzed strains do not present characteristics commonly related to pathogenicity in certain opportunistic microorganisms [39].

Another relevant aspect evaluated in this study was the antibiotic susceptibility profile. The analysis of antimicrobial resistance is a fundamental step in the characterization of microorganisms intended for probiotic or starter culture applications, since the presence of transferable resistance genes may represent a risk of dissemination of these genetic determinants in the environment or the intestinal microbiota. In this context, international guidelines, such as those established by the EFSA, emphasize the need for careful assessment of antimicrobial resistance [40].

The results indicated that the selected strains exhibited susceptibility profiles compatible with established safety criteria, since multidrug-resistant isolates were excluded from subsequent stages of the study. The specific evaluation of vancomycin resistance in coccoid isolates is also justified by the potential identification of *Enterococcus* spp., whose resistant strains represent a major public health concern, particularly in nosocomial infections [16].

Complementing the phenotypic tests, molecular analyses were performed to identify genes associated with virulence factors and beneficial traits in the studied strains. The results demonstrated that five of the thirteen analyzed strains presented at least one gene identified as a potential virulence factor. In parallel, genes frequently associated with probiotic potential were also investigated. Among them, the detection of the *map* gene in *Lpb. plantarum* 3 was noteworthy, as it is related to adhesion to the intestinal mucosa. This characteristic is considered relevant for probiotic microorganisms, since adhesion to the intestinal wall may favor temporary colonization of the gastrointestinal tract and enhance host–microbe interactions [21].

In addition to microbiological and safety characterization, the present study evaluated functional properties of the isolated strains, including antioxidant activity, survival under SDPS models, and exopolysaccharide (EPS) production. These properties are considered particularly important in the selection of microorganisms intended for functional food applications or as probiotic cultures [39].

The results regarding antioxidant activity indicated that the evaluated strains exhibited different levels of antioxidant capacity in the performed assays, including DPPH radical scavenging, metal ion chelation, hydroxyl radical scavenging, and inhibition of lipid peroxidation. The variability observed among strains reinforces the strain-dependent nature of these properties, a phenomenon widely reported in the literature for lactic acid bacteria.

The antioxidant activity of these bacteria may be associated with several biochemical mechanisms, including the production of antioxidant enzymes such as superoxide dismutase, the synthesis of metabolites with reducing activity, and the production of extracellular compounds such as EPS. In addition, certain peptides released during fermentation processes may also contribute to the neutralization of reactive oxygen species.

DPPH scavenging assays indicated high antioxidant activity in some of the analyzed strains, with values exceeding 90% in certain isolates. Such results suggest strong antioxidant potential and indicate that these strains may contribute to oxidative stress reduction when used in fermented food matrices. Additionally, the observed metal ion chelation activity, particularly for ferrous ions (Fe^2^⁺), represents an important antioxidant mechanism, since these ions can catalyze free radical formation through Fenton reactions [41].

On the other hand, hydroxyl radical scavenging results showed relatively lower values. This behavior may be related to differences in the neutralization mechanisms of distinct reactive oxygen species, since hydroxyl radicals exhibit extremely high reactivity and a very short half-life, which limits their neutralization by certain bacterial metabolites [41].

The ability to survive under SDPS conditions was also investigated, as this parameter is a fundamental criterion in the selection of potential probiotic microorganisms. The results demonstrated that most strains were able to survive the simulated stomach and duodenum conditions, indicating the presence of physiological mechanisms that allow tolerance to environmental stresses associated with the digestive system, such as acidic pH, digestive enzymes, and bile salts [40].

However, a reduction in antioxidant activity was observed after exposure to SDPS conditions. This result suggests that factors such as acidic pH, digestive enzymes, and bile salts may affect the stability or functional activity of certain metabolites produced by the strains. Nevertheless, the partial maintenance of antioxidant activity after exposure to adverse conditions represents a relevant characteristic for microorganisms intended for functional applications.

Another important result concerns EPS production by the evaluated strains. These extracellular polymers are of great interest in food microbiology and biotechnology, as they may act as thickening, stabilizing, and texturizing agents in fermented foods. In addition, EPS may also exhibit relevant biological properties, including antioxidants, immunomodulatory, and potential prebiotic activities [39].

Overall, the results indicate that the isolated strains exhibit promising characteristics for biotechnological applications. The combination of preliminary microbiological safety, antioxidant activity, survival under SDPS conditions, and EPS production suggests potential use of these bacteria in the development of functional foods, production of bioactive compounds, and as starter cultures in fermentation processes.

However, further studies are required to deepen the characterization of these strains, including whole-genome analyses, assessment of genetic stability, and in vivo studies to confirm their potential probiotic effects. In this context, the exploration of microorganisms isolated from natural matrices such as tropical fruits represents a promising strategy for the discovery of new strains with potential applications in food biotechnology and human health promotion.

Moreover, the production of these metabolites extends beyond microbial self-protection. Antioxidant molecules also favor the establishment of symbiotic interactions between LAB and their hosts by contributing to the reduction of oxidative stress in the intestinal tract. This process is directly related to the promotion of gut health and modulation of the immune response, configuring a mutualistic relationship in which both microorganisms and host benefit [42].

Within food systems, antioxidant metabolites produced by LAB play an important role in biopreservation. These compounds act by reducing lipid oxidation and maintaining the nutritional quality of products, contributing to shelf-life extension and reduced food waste.

In this context, the production of antioxidant metabolites by LAB represents an important adaptive mechanism. Such a mechanism not only enhances microbial survival but also strengthens host–microbe interactions and contributes to food stability. This set of functions highlights the relevance of LAB in health, nutrition, and sustainable food processing [43].

LAB antioxidant activity may occur through different mechanisms, including direct free radical scavenging, metal ion chelation that catalyzes ROS formation, and production of antioxidant enzymes such as superoxide dismutase (SOD) and glutathione peroxidase. In addition, metabolites produced by these microorganisms, such as bacteriocins and organic acids, also contribute to their antioxidant potential [4]. In food systems, these properties favor the preservation of quality and stability of fermented products by reducing lipid peroxidation processes and inhibiting oxidative reactions, thereby extending shelf life and enhancing nutritional benefits [6].

## Final considerations

This study aimed to isolate, characterize, and evaluate the functional potential of lactic acid bacteria (LAB) from fruits, considering microbiological safety, antioxidant activity, survival under SDPS models, and EPS production. Overall, the results demonstrated that fruits represent an important source of microorganisms with biotechnological and functional potential.

The isolation process revealed a diverse community of LAB, reflecting the microbial richness of natural plant matrices. This diversity is associated with the nutritional composition of fruits, particularly their high levels of sugars and organic compounds that favor the growth of fermentative microorganisms, reinforcing their role as natural reservoirs of biotechnologically relevant strains.

Regarding safety, the selected strains showed no hemolytic activity or production of enzymes associated with virulence, such as gelatinase and extracellular proteases, indicating a preliminary safe phenotypic profile for food applications. Nevertheless, molecular analyses are still required to confirm their safety at the genomic level.

The antioxidant assays demonstrated strain-dependent variability in free radical scavenging capacity and oxidative process reduction. In addition, several isolates were able to survive SDPS models, suggesting adaptive physiological mechanisms that support tolerance to gastric and intestinal stresses, an important trait for potential probiotic applications.

Moreover, the strains could produce EPS under different fermentation conditions, with yields influenced by the type of sugar used. These biopolymers are of considerable technological interest, as they may improve the functional and technological properties of fermented foods and may also contribute to antioxidant activity.

In conclusion, LAB isolated from fruits shows promising potential for biotechnological applications, particularly in the development of functional fermented foods. However, further studies, including genomic analyses and in vivo evaluations, are necessary to confirm their safety and functional efficacy for applications in food and health sectors.

## Supplementary Information

Below is the link to the electronic supplementary material.

Supplementary file1 (DOCX 39 KB)

## Data Availability

All data generated in this project is available from authors upon reasonable request.
