# Supplementary material for: Antioxidant properties of bioactive metabolites produced by lactic acid bacteria isolated from tropical fruits
Source: Braz J Microbiol. 2026 Aug 25;57(1):246. doi: 10.1007/s42770-026-02063-y (PMC13507007; doi:10.1007/s42770-026-02063-y)
Supplement: Supplementary file 1 [file 42770_2026_2063_MOESM1_ESM.docx]

**Supplementary material**

**Manuscript: “Antioxidant properties of bioactive metabolites produced by lactic acid bacteria isolated**

**from tropical fruits”**

**Table 1 – Isolated and identified strains evaluated for safety and antibiotic susceptibility**

| **Identification by 16S rRNA** | | ***Lmb. reuteri*** | ***Lmb. reuteri*** | ***Lab. pasteurii*** | ***Lpb. plantarum*** | ***Lmb. reuteri*** | ***Lmb. reuteri*** | ***Lmb. reuteri*** | ***Lpb. plantarum*** | ***Lmb. reuteri*** | ***L. lactis*** | ***Lpb. plantarum*** | ***Lvb. brevis*** | ***Strep. thermophilus*** |
| --- | --- | --- | --- | --- | --- | --- | --- | --- | --- | --- | --- | --- | --- | --- |
| **Strain code** | | G1 | G2 | G3 | G5 | K1 | Pi3 | Pi9 | Ce3 | Ce8 | Ce15 | 3 | 5 | 32 |
| **Origin** | | Guava (*goiaba)* | Guava (*goiaba)* | Guava (*goiaba)* | Guava (*goiaba)* | Cashew (*caju*) | Cherry (*pitanga*) | Cherry (*pitanga*) | Cherry  *(Cereja-do-rio-grande)* | Cherry  *(Cereja-do-rio-grande)* | Cherry  *(Cereja-do-rio-grande)* | Lemon (*limão cravo*) | Lemon (*limão cravo*) | Blackberry (*amora*) |
| **Standard tests** | Gram staining | + | + | + | + | + | + | + | + | + | + | + | + | + |
|  | Catalase | - | - | - | - | - | - | - | - | - | - | - | - | - |
|  | Gelatinase production | - | - | - | - | - | - | - | - | - | - | - | - | - |
|  | Mucin degradation | - | - | - | - | - | - | - | - | - | - | - | - | - |
|  | Hemolytic activity | - | - | - | - | - | - | - | - | - | - | - | - | - |
|  | Proteolytic activity | - | - | - | - | - | - | - | - | - | - | - | - | - |
| **Antibiotics, (µg/disco)** | Clorofenicol (30) | 20 | 25 | 30 | 32 | 18 | 22 | 30 | 36 | 23 | 15 | 34 | 22 | 23 |
|  | Streptomicin (10) | 25 | 0 | 28 | 30 | 13 | 12 | 17 | 32 | 40 | 18 | 30 | 26 | 20 |
|  | Gentamicin (10) | 30 | 15 | 20 | 27 | 26 | 5 | 10 | 15 | 20 | 23 | 0 | 38 | 20 |
|  | Tetraciclin (30) | 15 | 20 | 15 | 12 | 11 | 30 | 23 | 19 | 21 | 25 | 15 | 36 | 21 |
|  | Clindamicin (2) | 10 | 22 | 22 | 17 | 13 | 30 | 16 | 26 | 32 | 23 | 40 | 16 | 10 |
|  | Eritromicin (10) | 12 | 23 | 24 | 15 | 16 | 21 | 23 | 30 | 34 | 12 | 20 | 25 | 0 |
|  | Canamicin (30) | 0 | 0 | 10 | 23 | 32 | 11 | 20 | 13 | 15 | 30 | 33 | 32 | 24 |
|  | Tilosin (60) | 20 | 30 | 15 | 24 | 29 | 30 | 25 | 13 | 27 | 28 | 17 | 39 | 26 |
|  | Nalidix acid (30) | 0 | 0 | 10 | 22 | 0 | 5 | 10 | 11 | 5 | 0 | 0 | 17 | 10 |
|  | Vancomicin (30) | 22 | 5 | 5 | 10 | 8 | 11 | 21 | 12 | 10 | 9 | 32 | 29 | 29 |
|  | Ampicilin (10) | 25 | 10 | 0 | 5 | 4 | 5 | 0 | 5 | 6 | 0 | 40 | 40 | 0 |

| **Table 2 – Primers used for the detection of genes related to virulence, vancomycin resistance, adhesion, γ-aminobutyric acid (GABA) production, bile salt deconjugation, and biogenic amine production** | | | | | | | | | | | | | | | | | |
| --- | --- | --- | --- | --- | --- | --- | --- | --- | --- | --- | --- | --- | --- | --- | --- | --- | --- |
|  |  |  |  |  |  |  |  |  |  |  |  |  |  |  |  |  |  |
| **Genes** | **Description** | **Primer sequences (5′–3′)** | **PCR product size (bp)** | **Reference** | **Strains** | | | | | | | | | | | | |
| **Virulence genes** | | | | | **G1** | **G2** | **G3** | **G5** | **K1** | **Pi3** | **Pi9** | **Ce3** | **Ce8** | **Ce15** | **3** | **5** | **32** |
| *hyl* | Hyaluronidase | hyl F: 5′-ACAGAAGAGCTGCAGGAAATG-3′ | 276 | Vankerckhoven et al., 2004 |  |  |  |  |  |  |  |  |  |  |  |  | + |
|  |  | hyl R: 5′-GACTGACGTCCAAGTTTCCAA-3′ |  |  |  |  |  |  |  |  |  |  |  |  |  |  |  |
| *esp* | Enterococcal surface protein | esp14 F: 5′-AGATTTCATCTTTGATTCTTGG-3′ | 510 | Vankerckhoven et al., 2004 |  |  |  |  |  |  |  | + |  | + |  |  |  |
|  |  | esp12 R: 5′-AATTGATTCTTTAGCATCTGG-3′ |  |  |  |  |  |  |  |  |  |  |  |  |  |  |  |
| *IS16* | Pathogenicity island | IS16 F: 5′-CATGTTCCACGAACCAGAG-3′ | 547 | de Moraes et al., 2017 |  | + |  |  |  |  |  | + |  | + |  |  | + |
|  |  | IS16 R: 5′-TCAAAAAGTGGGCTTGGC-3′ |  |  |  |  |  |  |  |  |  |  |  |  |  |  |  |
| *efaA* | Endocarditis antigen | efa-A F: 5′-GCCAATTGGGACAGACCCTC-3′ | 688 | Martin-Platero et al., 2009 |  |  |  |  |  |  |  |  |  | + |  |  | + |
|  |  | efa-A R: 5′-CGCCTTCTGTTCCTTCTTTGGC-3′ |  |  |  |  |  |  |  |  |  |  |  |  |  |  |  |
| *cyt* | Cytolysin | cyt I F: 5′-ACTCGGGGATTGATAGGC-3 | 688 | Vankerckhoven et al., 2004 |  |  |  |  |  |  |  |  |  |  |  |  |  |
|  |  | cytIIb R: 5′-GCTGCTAAAGCTGCGCTT-3′ |  |  |  |  |  |  |  |  |  |  |  |  |  |  |  |
| *asa1* | Aggregation substance | asa1 F: 5′-GCACGCTATTACGAACTATGA-3′ | 375 | Vankerckhoven et al., 2004 |  |  |  |  |  |  |  |  |  |  |  |  |  |
|  |  | asa1 R: 5′-TAAGAAAGAACATCACCACGA-3′ |  |  |  |  |  |  |  |  |  |  |  |  |  |  |  |
| **Vancomycin resistance genes** | | | | | | | | | | | | | | | | | |
| *van*A | Vancomycin resistance | vanAB F: 5′-GTAGGCTGCGATATTCAAAGC-3′ | 230 | Valledor et al., 2020 |  |  |  |  |  |  |  |  |  |  |  |  |  |
|  |  | vanA R: 5′-CGATTCAATTGCGTAGTCCAA-3′ |  |  |  |  |  |  |  |  |  |  |  |  |  |  |  |
| *van*B | Vancomycin resistance | vanAB F: 5′-GTAGGCTGCGATATTCAAAGC-3′ | 300 | Valledor et al., 2020 |  |  |  |  |  |  |  |  |  |  |  |  |  |
|  |  | vanB R: 5′-GCCGACAATCAAATCCTC-3′ |  |  |  |  |  |  |  |  |  |  |  |  |  |  |  |
| *van*C | Vancomycin resistance | vanC F: 5′-ATCCAAGCTATTGACCCGCT-3′ | 360 | Valledor et al., 2020 |  |  |  |  |  |  |  |  |  |  |  |  |  |
|  |  | vanC R: 5′-TGTGGCAGGATCGTTTTCAT-3′ |  |  |  |  |  |  |  |  |  |  |  |  |  |  |  |
| *van*D | Vancomycin resistance | vanD F: 5′-TGTGGGATGCGATATTCAA-3′ | 500 | Valledor et al., 2020 |  |  |  |  |  |  |  |  |  |  |  |  |  |
|  |  | vanD R: 5′-TGCAGCCAAGTATCCGGTAA-3′ |  |  |  |  |  |  |  |  |  |  |  |  |  |  |  |
| *van*E | Vancomycin resistance | vanE F: 5′-TGTGGTATCGGAGCTGCAG-3′ | 510 | Valledor et al., 2020 |  |  |  |  |  |  |  |  |  |  |  |  |  |
|  |  | vanE R: 5′-GTCGATTCTCGCTAATCC-3′ |  |  |  |  |  |  |  |  |  |  |  |  |  |  |  |
| *van*G | Vancomycin resistance | vanG F: 5′-GAAGATGGTACTTTGCAGGGCA-3′ | 250 | Valledor et al., 2020 |  |  |  |  |  |  |  |  |  |  |  |  |  |
|  |  | vanG R: 5′-AGCCGCTTCTTGTATCCGTTTT-3′ |  |  |  |  |  |  |  |  |  |  |  |  |  |  |  |
| **Probiotic-related genes** | | | | | | | | | | | | | | | | | |
| *map* | Mucus adhesion | map F: 5′-TGGATTCTGCTTGAGGTAAG-3′ | 200 | Todorov and Dicks 2008 |  |  |  |  |  |  |  |  |  |  | **+** |  |  |
|  |  | map R: 5′-GACTAGTAATAACGCGACCG-3′ |  |  |  |  |  |  |  |  |  |  |  |  |  |  |  |
| *mub* | Mucus adhesion | mub F: 5′-GTAGTTACTCAGTGACGATCAATG-3′ | 200 | Todorov and Dicks 2008 |  |  |  |  |  |  |  |  |  |  |  |  |  |
|  |  | mub R: 5′-TAATTGTAAAGGTATAATCGGAGG-3′ |  |  |  |  |  |  |  |  |  |  |  |  |  |  |  |
| *eftu* | Adhesion-like factor | EFTu F: 5′-TTCTGGTCGTATCGATCGTG-3′ | 200 | Todorov and Dicks 2008 |  |  |  |  |  |  |  |  |  |  |  |  |  |
|  |  | EFTu R: 5′-CCACGTAATAACGCACCAAC-3′ |  |  |  |  |  |  |  |  |  |  |  |  |  |  |  |
| *prg*B | Aggregation substance | prgB F: 5′-GCCGTCGACTCGAGGAGAATGATACATGAAT-3′ | 3917 | Valledor et al., 2020 |  |  |  |  |  |  |  |  |  |  |  |  |  |
|  |  | prgB R: 5′-CCTGCGGCCGCGTCCTTCTTTTCGTCTTCAA-3′ |  |  |  |  |  |  |  |  |  |  |  |  |  |  |  |
| *EF*2662 | Choline-binding protein | EF2662 F: 5′-GGCGTCGACCACTTAAACTGATAGAGAGGAAT-3′ | 1121 | Valledor et al., 2020 |  |  |  |  |  |  |  |  |  |  |  |  |  |
|  |  | EF2662 R: 5′-CGCGCCGCAATTAATTATTAACTAGTTTCC-3′ |  |  |  |  |  |  |  |  |  |  |  |  |  |  |  |
| *EF*1249 | Fibronectin-binding protein | EF1249 F: 5′-GCGGTCGACAAACGAGGGATTTATG-3′ | 1712 | Valledor et al., 2020 |  |  |  |  |  |  |  |  |  |  |  |  |  |
|  |  | EF1249 R: 5′-CTGGCGGCCGCGTTTAATACAATTAGGAAGCAGA-3′ |  |  |  |  |  |  |  |  |  |  |  |  |  |  |  |
| *EF*2380 | Membrane-associated zinc metalloprotease | EF2380 F: 5′-GCGGTCGACATCTATGAAAACAAT-3′ | 1268 | Valledor et al., 2020 |  |  |  |  |  |  |  |  |  |  |  |  |  |
|  |  | EF2380 R: 5′-TCCGCGCCGCCTTAAACTTTCTCCTT-3′ |  |  |  |  |  |  |  |  |  |  |  |  |  |  |  |
| **Folate biosynthesis genes** | | | | | | | | | | | | | | | | | |
| *pab*B | Para-aminobenzoate synthase component I, ADCS | pabB F: 5′-CCTCAATTCATACAACCCTCTCACA-3′ | 1666 | Dos Santos et al., 2020 |  |  |  |  |  |  |  |  |  |  |  |  |  |
|  |  | pabB R: 5′-CAGACAAATCTTCACTCACGCCATAA-3′ |  |  |  |  |  |  |  |  |  |  |  |  |  |  |  |
| *pab*C | 4-Amino-4-deoxychorismate lyase, ADCL | pabC F: 5′-CGGACAAGCATAATGAATACTCGGAAT-3′ | 1850 | Dos Santos et al., 2020 |  |  |  |  |  |  |  |  |  |  |  |  |  |
|  |  | pabC R: 5′-GGATTGATAACCGCTTCTATTGCCGA-3′ |  |  |  |  |  |  |  |  |  |  |  |  |  |  |  |
| *fol*KQ | *fol*K, 2-amino-4-hydroxy-6-hydroxymethyl-dihydropteridine-diphosphokinase, HPPK; *fol*Q, dihydroneopterin-triphosphate-pyrophosphohydrolase | folK-Q F: 5′-CACTAGTGTCTATTGACTCAAATATTTT-3′ | 790 | Dos Santos et al., 2020 |  |  |  |  |  |  |  |  |  |  |  |  |  |
|  |  | folK-Q R: 5′-CGTTTTTATGGCTATCACGGGGCT-3' |  |  |  |  |  |  |  |  |  |  |  |  |  |  |  |
| *fol*PE | *fol*P, dihydropteroate synthase; *fol*E, GTPCyclohydrolase I | folP-E/F: 5′-GAGATAGTCTTAACGACATCACGATT-3′ | 1350 | Dos Santos et al., 2020 |  |  |  |  |  |  |  |  |  |  |  |  |  |
|  |  | folP-E/R: 5′-GCAGTCTATCAATTATTGGAAGCTTT-3′ |  |  |  |  |  |  |  |  |  |  |  |  |  |  |  |
| **GABA biosynthesis genes** | | | | | | | | | | | | | | | | | |
| *gad* | Glutamate decarboxylase production | cor F: 5′-CCTCGAGAAGCCGATCGCTTAGTTCG-3′ | 540 | Valledor et al., 2020 |  |  |  |  |  |  |  |  |  |  |  |  |  |
|  |  | cor R: 5′-TCATATTGACCGGTATAAGTGATGCCC-3′ |  |  |  |  |  |  |  |  |  |  |  |  |  |  |  |
| **Genes for Biogenic amines** | | | | | | | | | | | | | | | | | |
| *hdc*1 | Histidine decarboxylase | hdc1 F: 5′-AGATGGTATTGTTTCTTATG-3′ | 367 | De las Rivas et al., 2005 |  |  |  |  |  |  |  |  |  |  |  |  |  |
|  |  | hdc1 R: 5′-AGACCATACACCATAACCTT-3′ |  |  |  |  |  |  |  |  |  |  |  |  |  |  |  |
| *hdc*2 | Histidine decarboxylase | hdc2 F: 5′-AAYTCNTTYGAYTTYGARAARGARG-3′ | 534 | De las Rivas et al., 2005 |  |  |  |  |  |  |  |  |  |  |  |  |  |
|  |  | hdc2 R: 5′-ATNGGNGANCCDATCATYTTRTGNCC-3′ |  |  |  |  |  |  |  |  |  |  |  |  |  |  |  |
| *tdc* | Tyrosine decarboxylase | tdc F: 5′-GAYATNATNGGNATNGGNYTNGAYCARG-3′ | 924 | De las Rivas et al., 2005 |  |  |  |  |  |  |  |  |  |  |  |  |  |
|  |  | tdc R: 5′-CCRTARTCNGGNATAGCRAARTCNGTRTG-3′ |  |  |  |  |  |  |  |  |  |  |  |  |  |  |  |
| *odc* | Ornithine decarboxylase | odc F: 5′-GTNTTYAAYGCNGAYAARCANTAYTTYGT-3′ | 1446 | De las Rivas et al., 2005 |  |  |  |  |  |  |  |  |  |  |  |  |  |
|  |  | odc R: 5′-ATNGARTTNAGTTCRCAYTTYTCNGG-3′ |  |  |  |  |  |  |  |  |  |  |  |  |  |  |  |
